# Student perspective of classroom and distance learning during COVID-19 pandemic in the undergraduate dental study program Universitas Indonesia

**DOI:** 10.1186/s12909-020-02312-0

**Published:** 2020-10-29

**Authors:** Lisa R. Amir, Ira Tanti, Diah Ayu Maharani, Yuniardini Septorini Wimardhani, Vera Julia, Benso Sulijaya, Ria Puspitawati

**Affiliations:** 1 Dental Education Unit, Faculty of Dentistry, Universitas Indonesia, Jakarta, Indonesia; 2 Department of Oral Biology, Faculty of Dentistry, Universitas Indonesia, Jakarta, Indonesia; 3 Department of Prosthodontics, Faculty of Dentistry, Universitas Indonesia, Jakarta, Indonesia; 4 Department of Preventive and Public Health Dentistry, Faculty of Dentistry, Universitas Indonesia, Jakarta, Indonesia; 5 Department of Oral Medicine, Faculty of Dentistry, Universitas Indonesia, Jakarta, Indonesia; 6 Department of Oral and Maxillofacial Surgery, Faculty of Dentistry, Universitas Indonesia, Jakarta, Indonesia; 7 Department of Periodontology, Faculty of Dentistry, Universitas Indonesia, Jakarta, Indonesia

**Keywords:** Classroom learning, Distance learning, Blended learning, Undergraduate dental study program

## Abstract

**Background:**

The COVID-19 pandemic has become a global health issue and has had a major impact on education. Consequently, half way through the second semester of the academic year 2019/2020, learning methods were delivered through distance learning (DL). We aimed to evaluate the student perspective of DL compared to classroom learning (CL) in the undergraduate dentistry study program at the Faculty of Dentistry Universitas Indonesia.

**Methods:**

An online questionnaire was sent at the end of the semester. A total of 301 students participated in the study.

**Results:**

Duration of study influenced student preference. Higher number of first-year students preferred DL compared to their seniors (*p* < 0.001). Students preferred CL for group discussion, as DL resulted in more difficult communication and gave less learning satisfaction. Only 44.2% students preferred DL over CL, although they agreed that DL gave a more efficient learning method (52.6%), it provided more time to study (87.9%) and to review study materials (87.3%). Challenges during DL included external factors such as unstable internet connection, extra financial burden for the internet quota and internal factors such as time management and difficulty to focus while learning online for a longer period of time.

**Conclusion:**

Despite some challenges, dental students could adapt to the new learning methods of full DL and the majorities agreed blended learning that combined classroom and distance learning can be implemented henceforth. This current COVID-19 pandemic, changes not only the utilization of technology in education but the pedagogy strategies in the future.

**Supplementary information:**

**Supplementary information** accompanies this paper at 10.1186/s12909-020-02312-0.

## Background

The World Health Organization has declared the pandemic of the novel SARS-CoV2 infection early this year and it has now become a major public health challenge worldwide [1]. The infection control and physical distancing measures are crucial to prevent the virus from further spreading and to help control the pandemic situation. The policy of compulsory physical distancing has been implemented in many countries including in Indonesia [2, 3], resulting in nationwide school and university closures. In accordance with this policy, dental academic institutions are compelled to make appropriate and timely modification in order to continue to deliver education and to sustain the continuation of student academic progress. The teaching and learning activities were immediately shifted to a full E-learning.

E-learning is defined as learning that makes use of Information and Communication Technologies (ICTs). The incorporation of technological resources and innovative education strategies has transformed the teaching and learning processes. Previous studies have shown various e-learning and online learning tools that are effective for teaching and learning in the fields of health profession, including dentistry [4–8]. The knowledge gain and performance of the students as a result of E-learning were shown to be equivalent to that of face to face methods [9, 10]. Blended learning is mainly defined as the integration of classroom and distance learning to facilitate an independent, interactive and collaborative learning among students. However, to understand it in a more general perspective, blended learning approach redesign courses that are developed, scheduled and implemented through a combination of physical and virtual learning activities. It was previously reported that blended learning provides better student’s satisfaction, motivation, student engagement and performance [5, 7, 11, 12]. This approach promotes active and self-directed learning and has gained acceptance in dental education as a complementary method to traditional learning.

The undergraduate curriculum of the Faculty of Dentistry Universitas Indonesia adopted Student Centered Active Learning (SCAL) using collaborative learning, question-based learning or Problem-Based Learning (PBL) since 2003. In PBL, students work in groups to construct content knowledge and develop self-directed learning skills. The activities along the steps of the chosen learning methods (group discussions, clarification sessions, the laboratory works and skills lab) were all conducted in classroom learning with online support. The university E-learning management system (LMS) was utilized to facilitate various teaching and learning activities at different academic levels in the undergraduate dental program. The organization of courses, access to resources and additional learning materials are available through LMS to support self-directed learning within an integrated PBL curriculum. During this COVID-19 pandemic, courses delivered in student-centered learning methods were immediately moved to full E-learning. In the first half of semester before the pandemic, group discussions, clarification sessions and interactive lectures were carried out in-campus classroom learning while in the second half of semester, learning activities were delivered in full distance learning employing various online meeting platforms. In order to make the format of discussion sessions stay similar as it had been conducted before the pandemic, every online session was delivered synchronously with the attendance of a facilitator in each group. Students and facilitators’ time spent on setting or accomplishing tasks was similar as in classroom learning.

Despite previous reports on the comparison of classroom and distance learning [4–11], the evaluation on the student-centered active learning approaches that are delivered through blended learning methods compared to full online learning has not been widely available. The majorities of studies on distance learning method reported student perception of virtual learning modules that were integrated with classroom learning. Student feedback could provide important information for the evaluation of distance learning so as to improve future learning strategies. Therefore, the study aimed to analyze student perspective of SCAL delivered through full distance learning compared to the classroom learning in the undergraduate dentistry study program. An online questionnaire was distributed to the undergraduate dental students at the end of semester. We hypothesized students positive outcomes on the acceptance of distance learning as a new learning strategy that was implemented during COVID-19 pandemic condition.

## Methods

### Sampling procedures and participants

This study was performed from May to June 2020. Study participants were the first, second and third-year of undergraduate students of the dentistry study program at the Faculty of Dentistry Universitas Indonesia. The online questionnaire was given at the end of semester. They were strongly encouraged to fill out the questionnaire but their participation remained voluntary. The name and other personal information of the study participants were protected. Dental Research Ethics Committee Faculty of Dentistry Universitas Indonesia approved the study in accordance with the Helsinki Declaration (6/EA/FKGUI/VI/2020). Students were informed about the study and signed consent form.

### Learning methods

Before COVID-19 pandemic, learning strategies in the dentistry academic study program (pre-clinical) at the Faculty of Dentistry Universitas Indonesia was student-centered active learning. Collaborative learning (CL) and question-based learning (QBL) approaches were mainly used in the courses of the earlier semesters such as basic oral biology and introduction of health and dental science courses for the first-year dental students. Problem-based Learning (PBL) was mainly used in the courses of the latter semesters such as clinical dental science courses for the second and third-year dental students. The group discussions of these active learning approaches and lectures for clarification were delivered in classroom learning. Each group discussion consisted of 10–13 students and was supervised by 1 facilitator/tutor. Universitas Indonesia web-based education tools (EMAS, Moodle-based learning management system) was used to support various learning activities. Students could access the syllabus, learning objectives of each studied courses as well as scenarios/list of sub-topics or questions, list of references through the EMAS system and this learning approach represents blended learning.

As the COVID-19 pandemic protocol forced the compulsory work and study from home policy, since March 17, 2020, courses with CL, QBL and PBL methods were transferred to full distance learning. Group discussions, clarification lectures and assessments were carried out using various online platforms (Microsoft Teams, Google meets, Zoom and EMAS). Practice class and skills lab courses in which the expected learning outcomes involved various psychomotor skills were either substituted with video simulation, and or live and presented the stages of work online or postponed until the university is ready to be opened for the face-to-face classroom learning.

### Questionnaire

The questionnaire was developed to assess [1] the student’s perception of the distance learning method. The response options of the questionnaire items represent 4 Likert-type scales (0 = strongly disagree to 3 = strongly agree), except for questions of the most effective methods for distance learning (six options of the format of online learning) and open questions for the challenges and positive experience during distance learning. Altogether there were 22 statements in four parts: (A) general information on the student’s gender, year of study and GPA; (B) Preference; (C) Effectiveness, and; (D) Learning satisfaction.

### Statistical analysis

The internal consistency reliability questionnaire was measured by Cronbach’s alpha. Descriptive statistics were computed and bivariate analyses were performed. Logistic regression analyses were conducted to identify factors associated with the students’ preference towards distance learning. The level of statistical significance was at 0.05.

## Results

### General information

A total of 301 undergraduate first-, second- and third-year dental students of the Faculty of Dentistry Universitas Indonesia participated in the study. The response rate was 84.3%. Most of the participants were female (85.1%) and it reflects the majority of our undergraduate dental students (Table 1). Cronbach alpha of the questionnaire was 0.880. The Cronbach’s alpha coefficients of each domain were above 0.8, which was considered satisfactory. No CITC value was lower than 0.30, which allowed all items to be included in the instrument.

**Table 1:** General Information of the Study Participants (*n* = 301)

Variables	N (%)	Mean Preference Score (SD)	***P*** Value
Year of Study			
Class of 2017	90 (29.9%)	18.5 ± 5.7	0.001*
Class of 2018	97 (32.2%)	20.5 ± 6.1	
Class of 2019	114 (37.9%)	21.7 ± 5.5	
Gender			
Male	(45) 14.9%	20.4 ± 6.3	0.784**
Female	(256) 85.1%	20.3 ± 5.8	
GPA			
≤ 3.5	121 (40.2%)	20.7 ± 5.6	0.393***
≥ 3.51	180 (59.8%)	20.1 ± 6.0	

*Kruskal-Wallis, ** Mann-Whitney, *** T-test

### Preference domain

The total mean preference score was 20.3 ± 5.9, ranging from 2 to 36. Majorities of students (75.1%) agreed on the importance of classroom learning interaction for group discussion. Year of study influenced student’s perception toward distance learning. First-year students have a higher preference towards distance learning compared to their seniors (*p* < 0.001). There was no significant correlation between gender or grade point average (GPA) on students’ preference of learning methods (Table 1). Most students (87.4%) preferred synchronized learning sessions for group discussions and clarification sessions. Moreover, 58.8% students shared their concern on the online exams results, due to potential dishonesty of students.

### Effectiveness domain

Students perceived to have more learning time with the distance learning, although technical constraints still existed when doing distance learning (Table 2). Only 34.2% of students did not experience problems during distance learning. Data from open questions of the challenges during distance learning revealed the majority of the problems were categorized as external factors such as unstable internet connection and extra financial burden for internet quota. Other challenges related to internal factors included student readiness to the new learning method, time management and difficulties to focus while learning through the computer for a long period of time. These challenges might be contributed to the stress experienced by 35.2% students during distance learning (Table 2).

**Table 2:** The Percentage of Dental Students’ Agreement with the Statements Given on Distance Learning

Statements		Likert Score				
		Strongly Disagree	Disagree	Agree	Strongly Agree	Domain Mean Preference ±SD
**B. Preference Domain**						**1.89 ± 0.58**
1	Clarification sessions is more suitable delivered in distance learning	1.33%	23.59%	53.49%	21.59%	
2	Assessment is more suitable delivered in distance learning	1.66%	28.24%	55.48%	14.62%	
**C. Effectiveness Domain**						**1.84 ± 0.56**
3	I do not experience any problems during distance learning	11.63%	54.15%	28.24%	5.98%	
4	I do not experience stress during distance learning	5.98%	29.24%	45.18%	19.60%	
5	I have more time to prepare learning materials before group discussion with distance learning	2.66%	9.63%	57.48%	30.23%	
6	I have more time to review all of the learning materials after class with distance learning	2.33%	10.63%	59.14%	27.90%	
**D. Learning Satisfaction Domain**						**1.53 ± 0.59**
7	Distance learning give similar learning satisfaction than classroom learning	10.30%	51.49%	33.89%	4.32%	
8	Distance learning can be implemented in the next semester	4.65%	30.56%	52.49%	12.30%	
9	Distance learning give motivation for self directed learning and eager to prepare learning materials before group discussion	5.65%	32.56%	47.51%	14.28%	
10	Communication with lecturers and fellow students is easier with distance learning	6.31%	53.49%	30.23%	9.97%	
11	I like distance learning than classroom learning	10.30%	45.51%	34.22%	9.97%	
12	I study more efficiently with distance learning	6.64%	41.20%	39.53%	12.63%	

The responses to each of 12 statements were scored using a Likert scale ranging from 0 to 3 (strongly disagree, disagree, agree, and strongly agree)

### Learning satisfaction domain

The results of logistic regression confirmed the suitability, preferability, communication, sustainability, efficiency, satisfaction and motivation were significant factors related to the students’ preference towards distance learning (Table 3). Overall, efficiency has the highest odds ratio in relation to preference towards distance learning. However, 61.7% students disagreed that distance learning gave similar learning satisfaction to classroom learning.

**Table 3:** Variables Related to the Students’ Preference Toward Distance Learning (Logistic Regression)

Variable	Odds ratio	95% CI	***p***-value
Efficient	18.0	4.5–72.8	0.000
Sustainable	17.7	3.4–91.4	0.001
Likeable	9.3	3.2–26.6	0.000
Motivating	7.8	1.8–34.4	0.006
Less constraint	4.5	1.9–10.7	0.001
Grades	4.1	1.6–10.6	0.004
Suitable for exam	4.0	1.3–12.1	0.014
Good communication	2.4	1.0–5.7	0.044

*CI* confidence interval

### Correlation

The correlations between each 12 variables were shown in Table 4. Item sub-scale correlations ranged from 0.140–0.763, indicating the multidimensionality of the questionnaire scale. Strong correlation was observed between sufficient time to prepare lessons and sufficient time to review the study materials in distance learning and efficiency related to motivation. Correlations were all significant at the *p* < 0.05 level.

**Table 4:** Associations (Spearman’s correlations) between 12-items of the Preference Scale

	Suitable for Lecture	Suitable for Exam	Less Constraint	Unstressful	Sufficient Learning Time	Sufficient Review Time	Good Communication	Likeable	Sustainable	Efficient	Satisfying	Motivating
**Suitable for Lecture**	1											
**Suitable for Exam**	0.368	1										
**Less Constraints**	0.164	0.140	1									
**Unstressful**	0.258	0.169	0.477	1								
**Sufficient Learning Time**	0.321	0.172	0.258	0.378	1							
**Sufficient Review Time**	0.360	0.184	0.267	0.444	**0.763**	1						
**Good Communication**	0.212	0.212	0.136	0.194	0.195	0.207	1					
**Likeable**	0.315	0.281	0.325	0.508	0.450	0.442	0.382	1				
**Sustainable**	0.403	0.305	0.240	0.333	0.454	0.374	0.333	0.522	1			
**Efficient**	0.340	0.266	0.315	0.393	0.544	0.490	0.424	**0.603**	0.540	1		
**Satisfaction**	0.156	0.190	0.313	0.361	0.297	0.254	0.305	0.515	0.433	0.530	1	
**Motivation**	0.323	0.257	0.277	0.365	0.515	0.436	0.388	0.542	0.584	**0.672**	0.566	1

Correlations were all significant at *p* < 0.05

## Discussion

The COVID-19 pandemic has brought the unprecedented universities’s facilities closure, it affected millions of students worldwide. The sudden transformation in the teaching and learning activities into virtual modalities was carried out in order to continue the academic courses while avoiding people gathering and the potential risk of the spread of infection. The present study documented the student perspective of student-centered active learning delivered through full distance learning since March 17, 2020 and compared to the classroom learning in the undergraduate dentistry study program. Full distance learning whereby group discussions were carried out synchronously through the online communication platforms is a new learning method that has not been previously implemented in our dental school. This study was the first to compare the student perceptions on both types of learning methods related to the preference, effectiveness and learning satisfaction reported during the COVID-19 pandemic condition.

The survey demonstrated 44.2% students preferred distance learning over classroom learning. This result was lower than other studies comparing online and traditional learning methods which reported higher preference toward e-learning compared to traditional classroom methods [5, 13, 14]. Student’s attitude and acceptance toward e-learning has been shown to be more positive and favorable. However, in these studies the virtual learning modules were integrated with classroom learning, while in the present study, the distance learning was delivered in full online. It was previously reported that full online learning offers a sense of unreality and it largely depends on the students commitment to the courses [15]. Bridges and colleagues suggested the integration of learning technologies with face-to-face teaching to support access to digital resources and to enhance the visualization [16]. Blended PBL structured similarly as traditional PBL while offering the ability to use online communication tools and online environment to share materials. These differences in the learning methods and the new learning strategy experienced by our dental students might explain the lower percentage of students preferred full distance learning observed in this study.

In this study, the preference on learning methods was influenced by the year of study. Among students who preferred distance learning, the percentage of freshman students was significantly higher than the seniors. Similarly, studies conducted by Sritongthaworn et al. (2006) and Teo at al (2011) reported that younger students tend to adapt to e-learning [17, 18]. One of the factors that contribute to this finding might be related to the curriculum implemented at the time of this study. Senior dental students learned more clinical dental science courses which involve both theory and procedural knowledge and skills. Essentially such courses require laboratory skill sessions to enhance the understanding of the learned subjects. As the execution of dental laboratory works and practical was postponed due to the university closure, this resulted in the lack of motoric skills experiences, less chance of direct consultation with the instructors and less practical assignments that were normally served as the reinforcement to the theory class. While the curriculum of first-year dental students studied more basic dental science courses which are mostly conceptual theories so that the content knowledge acquisition could still be re-enforced by laboratory activities based on online tutorial and exercises in form of video or photographs. It is well comprehended that dental education can not be carried on the same way as medical education. The reason of this difference is that the dental students requires adequate physical setting and psychomotor skills, even since in the academic years, and that could not be replaced by distance learning strategy as being conducted during the pandemic [2].

Beside the necessary preparedness of students in distance learning methods, other factors such as personality types may influence student preference for e-learning than classroom learning [19–21]. As the personality regulates how individuals perceive, make judgements and react in certain situations. The acceptance of students for e-learning is commonly associated with self regulation character. Self regulatory behavior includes the ability to set goals, effective time management, problem solving capacity, and awareness of time to seek advice from instructors [20–22]. On top of self regulatory behavior, constraint of self efficacy, e-learning motivation, and high task value are other factors which strengthen the blended/online learning preference [21, 22]. It is interesting to note that despite the lower percentage of distance learning preference observed in this study, students agreed that distance learning could motivate them to prepare the learning materials before group discussion.

Logistic regression analysis confirmed efficiency has the highest odds ratio in relation to preference towards distance learning. Moreover, students recognized there was more time to study and to review study material in distance learning. Such results are in line with previous studies which has been demonstrated that distance learning offers higher flexibility of place of study process, saving time and cost since commuting from and to campus is no longer needed [23]. Well designed distance learning gives more time for students to access more topics and unlimited information. Such advantage suits the learning process of medical and dental students in recent decades since they have to digest increased loads of new and kept updated topics [5].

Apart from its obvious advantages, distance learning also brings some disadvantages. Increased chances of distraction, complicated technology, limited social interaction, and increased difficulty to stay in contact with instructors are several conditions that might interfere with the success of distance learning [24]. The present study showed more students felt lower learning satisfaction and more difficult communication either with instructors or with peer students in doing distance learning. Internal factors challenges of student readiness to distance learning, time management and difficulty to stay focused for long online learning duration were reported. Besides the students internal factor as mentioned above, other categories of distance learning barriers were also present in the time and environment when this study was conducted. The performance of instructors in charge in the distance learning process of this study were varied in their interactive pedagogy ability, uplifting spirit, and confidence toward utilization of innovative learning. Self efficacy character is importantly demanded not only from students but also from instructors. The quality of teaching is very important in stimulating students’ satisfaction. Special attention to communicate with students is essential since lack of personal contact may affect the development of trust [22, 23]. Peer to peer communication and interaction in a group discussion are not often feasible in the virtual learning method. The barriers associated with infra-structure were obviously also encountered by the students complaining about unstable internet connection and extra financial burden for internet quota. Moreover, stress experienced by one-third of the participants of the study might have an impact on student perspective toward learning method. Recent study also reported students concerned on the issues of economic slowdown, potential academic delay and changes in daily life and these were associated with the level of anxiety of the college student in China during this pandemic time [24].

The present study demonstrated important findings that are essential for the improvement and development of learning strategies in the future. However, this study had some limitations. First, the generalizability of the study was limited by the use of data from a single university. Second, although students were encouraged to take part in this study, their participation was voluntary. The response rate of 84.3% was below the 90% response rate that was initially targeted. The number of non-respondents may therefore have undermined the power of the study and the potential response bias can not be completely ruled out [25]. The results of the study must therefore be interpreted with caution. Third, the study focused on the preclinical students as its respondents, while the more challenging adaptation in learning strategy in dentistry during the pandemic is critically faced by the clinical students in the profession program. Forth, the questionnaire used in this study only measured student perception. It was unclear how student academic performance was affected by distance learning strategy, whether there were any difficulties encountered by students in understanding course learning outcomes, particularly for senior-year students who received clinical dental science courses and have lower preference toward distance learning. Previously, it was reported a weak correlation between the student perception of learning with the actual gain of knowledge [26]. Student perception may not reflect student understanding of course learning outcomes. Therefore, assessing the impact of distance learning on student academic performance is as crucial for the evaluation of curriculum transformation. This should be further investigated. Despite these limitations, the results of this study offer valuable information on the current perspectives of dental students with regard to full distance learning methods implemented during the COVID-19 pandemic. As student acceptance of learning method play an important role in creating an effective learning environment [27, 28]. Due to the uncertainty in this COVID-19 pandemic time, whereby the situation is still changes, it is essential to design the learning method that is most suited to current situation and to have appropriate plan once it is permissible for classroom teaching to resume its activities, taken into consideration all the necessary protocols for safety and health protection [29].

## Conclusion

The study presented evidence that despite some challenges, undergraduate dental students could adapt to the new learning methods of distance learning and agreed on better efficiency experienced in distance learning than in classroom learning. This sudden closure of the university globally due to COVID-19 pandemic, albeit undesirable, presents an enormous opportunity for cultural transformation in the education system. As more “tech-savvy” generations enroll in higher education, dental educators need to incorporate blended learning in the curriculum, to design the best features of classroom and distance learning to improve the overall learning environment.

## Supplementary information

**Additional file 1.**

## Data Availability

All of the relevant raw data of this study will be available from Ria Puspitawati (corresponding author) for scientists who wish to use them for non-commercial purposes.

## Abbreviations

CITC: Corrected Item Total Corrections
CL: Classroom Learning
DL: Distance Learning
EMAS: E-learning management system
PBL: Problem-based Learning
QBL: Question-Based Learning
SCAL: Student Centered Active Learning
