# Supplementary material for: Student perspective of classroom and distance learning during COVID-19 pandemic in the undergraduate dental study program Universitas Indonesia
Source: BMC Med Educ. 2020 Oct 29;20:392. doi: 10.1186/s12909-020-02312-0 (PMC7594975; doi:10.1186/s12909-020-02312-0)
Supplement: Supplementary file 1 — Additional file 1. [file 12909_2020_2312_MOESM1_ESM.pdf]

Questionnaire

STUDENT PERSPECTIVE OF CLASSROOM AND DISTANCE LEARNING METHOD DURING COVID-19 PANDEMIC IN THE UNDERGRADUATE DENTAL STUDY PROGRAM

Manuscript ID: MEED-D-20-00904

==

1. Year of Study*
   - 2016
   - 2017
   - 2018
   - 2019 [Regular Class]
   - 2019 [International Class]

2. Gender*
   - Male
   - Female

3. Grade Point Average (GPA) score*
   (short answer)___

4. Group discussion [QBL/CL/PBL] is more suitable delivered in classroom learning*
   - Strongly agree
   - Agree
   - Disagree
   - Strongly disagree

5. Clarification session is more suitable delivered in distance learning*
   - Strongly agree
   - Agree
   - Disagree
   - Strongly disagree

6. Assessment is more suitable delivered in distance learning*
   - Strongly agree
   - Agree
   - Disagree
   - Strongly disagree

7. I do not experience stress during distance learning *
   - Strongly agree
   - Agree
   - Disagree
   - Strongly disagree

8. I do not experience any problems during distance learning*
- Strongly agree
- Agree
- Disagree
- Strongly disagree

9. I have more time to prepare learning materials before group discussion with distance learning*
- Strongly agree
- Agree
- Disagree
- Strongly disagree

10. I have more time to review all of the learning materials after class with distance learning*
- Strongly agree
- Agree
- Disagree
- Strongly disagree

11. Distance learning give similar learning satisfaction than classroom learning*
- Strongly agree
- Agree
- Disagree
- Strongly disagree

12. Blended learning (combination of classroom and distance learning) can be implemented in the next semester*
- Strongly agree
- Agree
- Disagree
- Strongly disagree

13. Distance learning give motivation for self directed learning and eager to prepare learning materials before group discussion*
- Strongly agree
- Agree
- Disagree
- Strongly disagree

14. Communication with lecturers and fellow students is easier with distance learning*
- Strongly agree
- Agree
- Disagree
- Strongly disagree

15. I like distance learning more than classroom learning *
- Strongly agree
- Agree

- Disagree
- Strongly disagree

16. I study more efficiently with distance learning*
    - Strongly agree
    - Agree
    - Disagree
    - Strongly disagree

17. Distance learning gives the opportunity for students to cheat during assessment*
    - Strongly agree
    - Agree
    - Disagree
    - Strongly disagree

18. The most effective method for group discussion*
    - Synchronous, audio-video-text based
    - Synchronous, audio-text based
    - Synchronous, text based
    - Asynchronous, audio-video-text based
    - Asynchronous, audio-text based
    - Asynchronous, text based

19. The most effective method for group clarification session*
    - Synchronous, audio-video-text based
    - Synchronous, audio-text based
    - Synchronous, text based
    - Asynchronous, audio-video-text based
    - Asynchronous, audio-text based
    - Asynchronous, text based

20. The most effective method for group interactive lectures*
    - Synchronous, audio-video-text based
    - Synchronous, audio-text based
    - Synchronous, text based
    - Asynchronous, audio-video-text based
    - Asynchronous, audio-text based
    - Asynchronous, text based

21. Challenges experienced during distance learning*

Paragraph answer____

22. Positive aspects of distance learning*

Paragraph answer____
